# Self-assembled molecular hybrids comprising lacunary polyoxometalates and multidentate imidazole ligands†

**DOI:** 10.1039/d4sc02384f

**Published:** 2024-05-16

**Authors:** Haoran Sun, Atsuhiro Jimbo, Chifeng Li, Kentaro Yonesato, Kazuya Yamaguchi, Kosuke Suzuki

**Affiliations:** a Department of Applied Chemistry, School of Engineering, The University of Tokyo 7-3-1 Hongo, Bunkyo-ku Tokyo 113-8656 Japan ksuzuki@appchem.t.u-tokyo.ac.jp

## Abstract

Self-assembly *via* coordination bonding facilitates the creation of diverse inorganic–organic molecular hybrids with distinct structures and properties. Recent advances in this field have been driven by the versatility of organic ligands and inorganic units. Lacunary polyoxometalates are a class of well-defined metal-oxide clusters with a customizable number of reactive sites and bond directions, which make them promising inorganic units for self-assembled molecular hybrids. Herein, we report a novel synthesis method for self-assembled molecular hybrids utilizing the reversible coordination of multidentate imidazole ligands to the vacant sites of lacunary polyoxometalates. We synthesized self-assembled molecular hybrids including monomer, dimers, and tetramer, demonstrating the potential of our method for constructing intricate hybrids with tailored properties and functionalities.

## Abstract 2

This work presents a synthesis method for self-assembled molecular hybrids utilizing the reversible coordination of multidentate imidazole ligands to the vacant sites of lacunary polyoxometalates.

## Graphical abstract

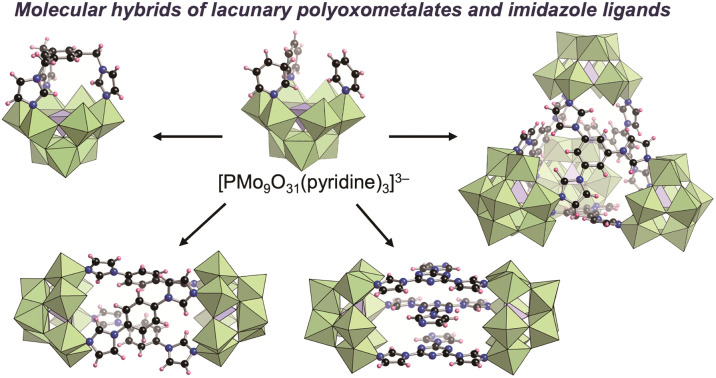

## Introduction

Self-assembly *via* coordination bonding is a powerful approach for constructing various inorganic–organic molecular hybrids with interesting applications such as catalysis, reactivity control, molecular recognition, and separation.^1^ Recent remarkable advances in this field have been enabled by the diversity and design possibilities of both organic ligands and inorganic units. Polyoxometalates (POMs) are a class of structurally well-defined inorganic metal-oxide clusters, where metals are typically in high oxidation states such as Mo^6+^, W^6+^, and V^5+^.^2^ Their structures and constituent elements can be tailored to facilitate the modification of their chemical and physical properties for various applications, such as in catalysis, photocatalysis, sensors, batteries, and energy conversion.^2^ Integrating organic ligands into POM systems enables further control over their stabilities, structures, and functionalities.^3,4^ In particular, lacunary POMs, where one or more [MO_*x*_] units are removed from the plenary structure, possess vacant reactive sites for metal atoms or organic ligands.^2,5^ Moreover, the number of reactive sites and bond directions can be tailored depending on the lacunary structure; therefore, these lacunary POMs are promising inorganic units for constructing coordination-driven inorganic–organic molecular hybrids. Various types of organic ligands such as phosphates, amines, silanols, and alcohols have been used for constructing POM–organic hybrids *via* coordination with the vacant sites of lacunary POMs^3,6^ or additionally incorporated metal sites at these vacant sites.^7^

Recently, our research group reported that pyridine-based ligands are effective for constructing POM–organic hybrids *via* reversible coordination bonds between pyridine and the vacant sites of lacunary POMs.^8^ Three pyridine molecules can coordinate with the Mo atoms at the vacant sites of a trivacant lacunary Keggin-type phosphomolybdate (TBA_3_H_6_[A-α-PMo_9_O_34_], TBA = tetra-*n*-butylammonium) to form TBA_3_[A-α-PMo_9_O_31_(pyridine)_3_]. Moreover, we found that this pyridine-coordinated hybrid can be reacted with multidentate pyridine-based ligands in organic solvents to synthesize more complex molecular hybrids *via* ligand exchange reactions.^8^

We hypothesized that imidazole is a promising ligand to expand this approach because it has a stronger coordination ability with metal ions compared with pyridine and its five-membered ring structure offers coordination directions that differ from the six-membered ring structure of pyridine-based ligands.^9^ To date, various imidazole-based metal–organic structures such as cages and capsules have been synthesized using metal ions and imidazole ligands as building units.^10^ Although several hybrids of POMs and imidazole ligands synthesized *via* hydrogen bonding and electronic interaction as well as coordination to the substituted metals in POMs have been reported,^11^ a system directly coordinating imidazole to lacunary POMs has not yet been reported.

**Fig. 1:**
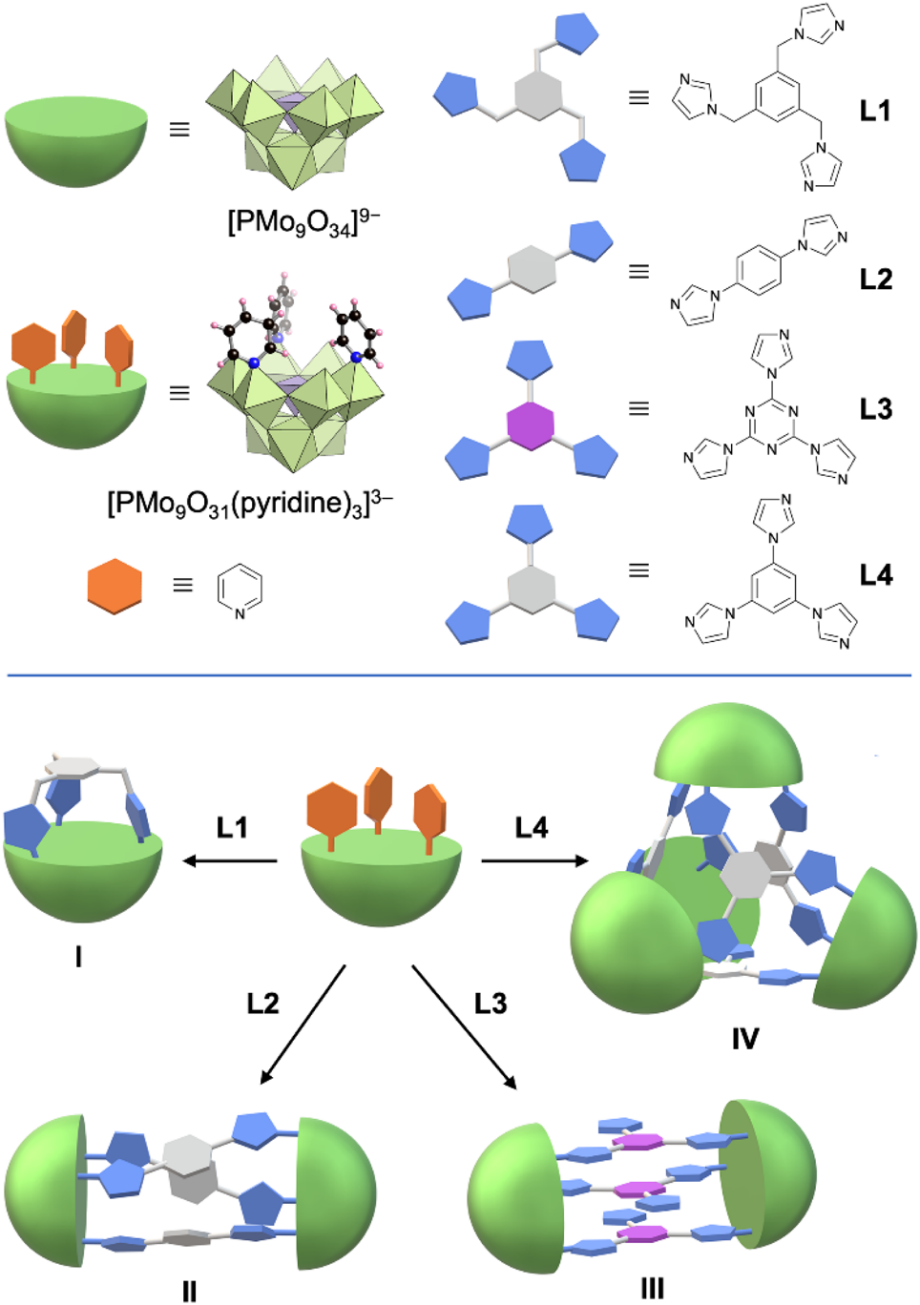
Self-assembly of molecular hybrids I, II, III, and IV using multidentate imidazole ligands and pyridine-coordinated trivacant lacunary phosphomolybdate.

Herein, for the first time, we present a synthesis method for fabricating self-assembled molecular hybrids comprising lacunary POMs and multidentate imidazole ligands. By reacting a pyridine-coordinated trivacant lacunary phosphomolybdate TBA_3_[A-α-PMo_9_O_31_(pyridine)_3_] with four types of multidentate imidazole ligands in organic solvents, we successfully synthesized a series of self-assembled molecular POM–organic hybrids: a capped monomer I, pillared dimer II, sandwich-like dimer III, and tetrahedron tetramer IV through the direct coordination of imidazole ligands to lacunary POMs (Fig. 1).

## Results and discussion

**Fig. 2:**
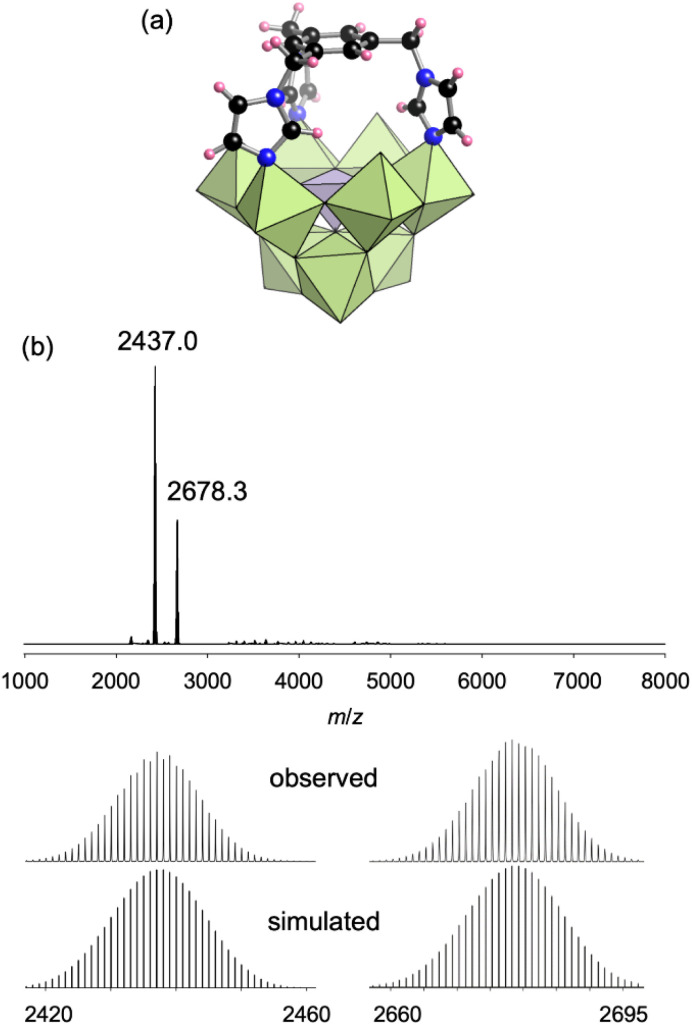
(a) Crystal structure of the anion part of I. Light-green and light-purple polyhedra represent [MoO_6_] and [PO_4_], respectively. Blue, black, and pink spheres represent N, C, and H atoms, respectively. (b) ESI mass spectrum of I in acetonitrile. Inset: enlarged spectra and simulated patterns for *m*/*z* 2437.0 ([TBA_3_H(PMo_9_O_31_)(L1)]^+^) and 2678.3 ([TBA_4_(PMo_9_O_31_)(L1)]^+^).

To synthesize I, 1,3,5-tris[(1*H*-imidazol-1-yl)methyl]benzene (L1), which possesses three imidazole groups on a benzene ring through flexible methylene groups, was used as a ligand and reacted with TBA_3_[A-α-PMo_9_O_31_(pyridine)_3_] in acetonitrile at 80 °C for 10 min (L1 : TBA_3_[A-α-PMo_9_O_31_(pyridine)_3_] = 2 : 1). This was followed by recrystallization in a mixed solvent of 1,2-dichloroethane and diethyl ether, which yielded single crystals of I. X-ray crystallographic analysis revealed that the anion part of I adopted a capped monomer structure comprising one [α-PMo_9_] unit and one L1 ligand, wherein three imidazole groups of L1 coordinated to three Mo atoms at the vacant site of the [α-PMo_9_] unit (Fig. 2a, Table S1†). The electrospray ionization (ESI) mass spectrum of I in acetonitrile exhibited sets of signals at *m*/*z* 2437.0 and 2678.3, which were attributed to [TBA_3_H(PMo_9_O_31_)(L1)]^+^ and [TBA_4_(PMo_9_O_31_)(L1)]^+^, respectively (Fig. 2b). The ^31^P nuclear magnetic resonance (NMR) spectrum of I in acetonitrile-*d*_3_ (CD_3_CN) exhibited a signal at −2.06 ppm (Fig. S1a†), which was observed up-field compared with TBA_3_[A-α-PMo_9_O_31_(pyridine)_3_] (−0.68 ppm).^8*a*^ The ^1^H NMR spectrum of I in CD_3_CN showed the splitting of the signals of the methylene H atoms in the ligand at 5.11 ppm (d, *J* = 13.5 Hz) and 4.88 ppm (d, *J* = 13.5 Hz), which can be ascribed to the geminal H–H spin–spin coupling on the same methylene C atom (Fig. S1b†). This observation of ^1^H NMR spectrum agrees with the rigid structure of I, where two H atoms in a methylene group exist in different chemical environments. We also confirmed that the ^31^P NMR spectrum remained almost unchanged after 3 days, demonstrating the stability of I in the solvent (Fig. S1c†). Based on these results as well as elemental analysis and thermogravimetry differential thermal analysis (TG-DTA) results (Fig. S2a†), the formula of I was determined to be TBA_3_[(PMo_9_O_31_)(L1)](C_2_H_4_Cl_2_)_2_.

**Fig. 3:**
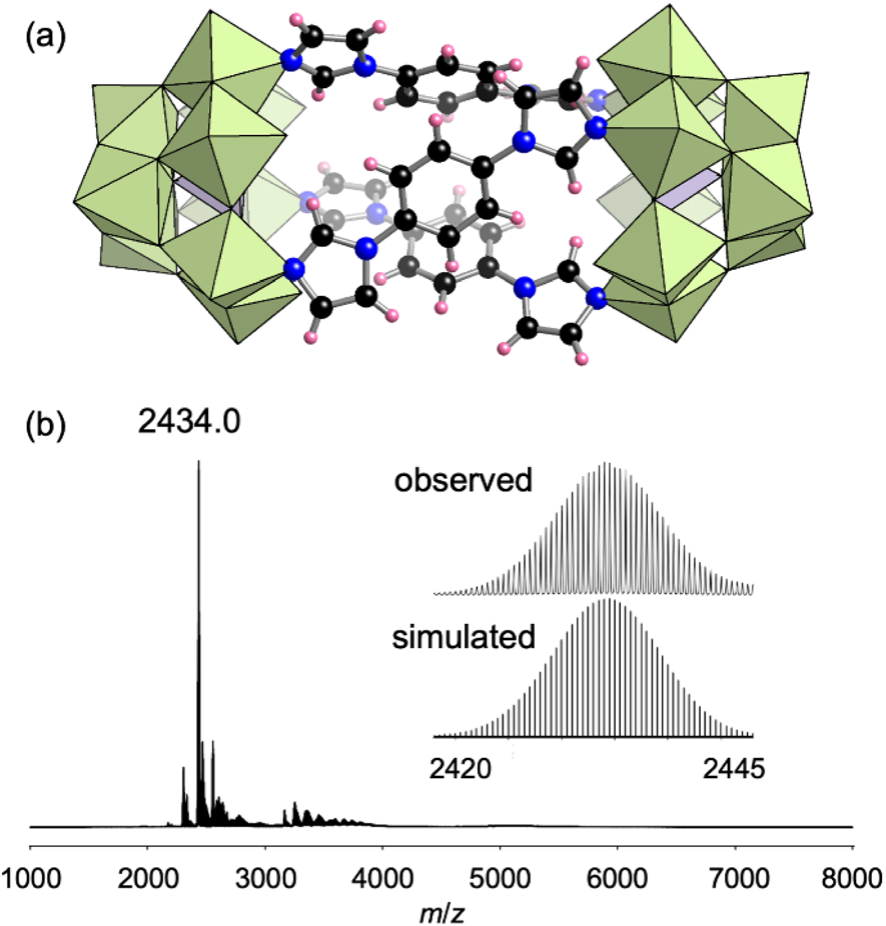
(a) Crystal structure of the anion part of II. (b) ESI mass spectrum of II in acetonitrile. (Inset): enlarged spectrum and simulated pattern for *m*/*z* 2434.0 ([TBA_6_H_2_(PMo_9_O_31_)_2_(L2)_3_]^2+^).

To construct oligomeric hybrid structures, we investigated the use of rigid multidentate ligands. TBA_3_[A-α-PMo_9_O_31_(pyridine)_3_] was reacted with 1,4-di(1*H*-imidazol-1-yl)benzene (L2), which is a linear ligand possessing two imidazole groups, in 3 : 1 ratio in acetonitrile at 50 °C for 10 min. By recrystallization of the crude product in 1,2-dichloroethane, single crystals of II suitable for X-ray crystallographic analysis were obtained. The anion part of II had a pillared dimer structure where two [PMo_9_] units were bridged by three L2 ligands (Fig. 3a, Table S1†). Some of the [α-PMo_9_] units were transformed into [β-PMo_9_] units *via* rotation of the [Mo_3_O_13_] unit underneath by approximately 60° from the original orientation (Fig. S3†). X-ray crystallographic analysis results showed that the ratio of [α-PMo_9_] and [β-PMo_9_] was 1 : 0.07. The ^31^P NMR spectrum of II in CD_3_CN exhibited two signals at −1.34 and −0.01 ppm with a signal integral ratio of 1 : 0.09, which were attributed to the [α-PMo_9_] and [β-PMo_9_] isomers, respectively (Fig. S4†). The ESI mass spectrum of II in acetonitrile exhibited a set of signals at *m*/*z* = 2434.0, which was assignable to [TBA_6_H_2_(PMo_9_O_31_)_2_(L2)_3_]^2+^ (Fig. 3b). These NMR and ESI mass spectra showed that II maintained its structure in the solvent. The ^31^P NMR spectrum of II in CD_3_CN after 3 days showed only two signals at −1.34 and −0.01 ppm, but the integral ratio of these signals changed from 1 : 0.09 to 1 : 0.27 (Fig. S4c†). This result showed that although the ratio of [α-PMo_9_] and [β-PMo_9_] in II changes after several days, the structure of II is stable and kept in the solvent. Based on these results as well as the elemental analysis and TG-DTA results (Fig. S2b†), the formula of II was determined to be TBA_5.7_H_0.3_[(PMo_9_O_31_)_2_(L2)_3_](C_2_H_4_Cl_2_).

**Fig. 4:**
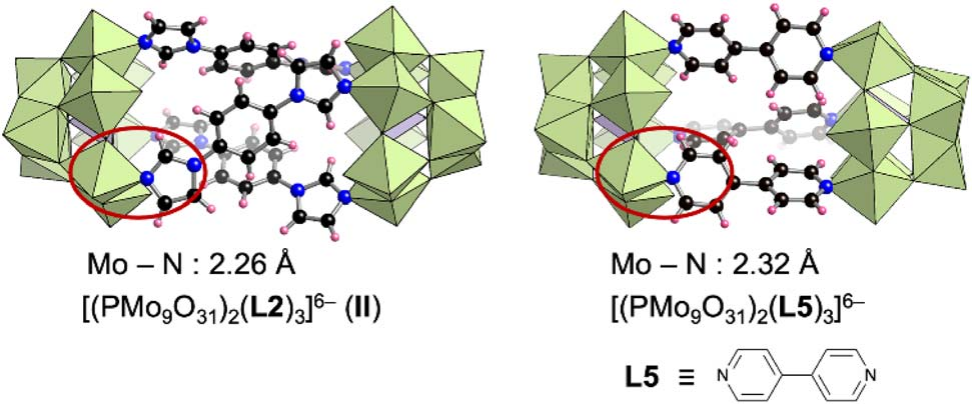
Structures of [(PMo_9_O_31_)_2_(L2)_3_]^6−^ (II) and [(PMo_9_O_31_)_2_(L5)_3_]^6−^ and their average Mo–N bond lengths.^8*a*^

The structure of II is similar to that of our previously reported hybrid dimer comprising three 4,4′-bipyridine ligands (L5) and two [PMo_9_] units ([(A-α-PMo_9_O_31_)_2_(L5)_3_]^6−^) (Fig. 4).^8*a*^ Changing the pyridine-based ligand to the imidazole-based ligand shortened the Mo–N bond lengths in II (2.26 Å on average, Mo–imidazole coordination bonds) compared with those in [(A-α-PMo_9_O_31_)_2_(L5)_3_]^6−^ (2.32 Å on average, Mo–pyridine coordination bonds) (Fig. 4). Although only [α-PMo_9_] units were observed in [(A-α-PMo_9_O_31_)_2_(L5)_3_]^6−^, isomerized [β-PMo_9_] units were also observed in II. To investigate the formation of these imidazole- and pyridine-based hybrids as well as the isomerization of [α-PMo_9_] to [β-PMo_9_] units, we performed density functional theory calculations for these reactions. The standard Gibbs energy of the reaction 2[A-α-PMo_9_O_31_(pyridine)_3_]^3−^ + 3L2 → [(A-α-PMo_9_O_31_)_2_(L2)_3_]^6−^ (II) + 3pyridine was −69.2 kJ mol^−1^, while that for the reaction 2[A-α-PMo_9_O_31_(pyridine)_3_]^3−^ + 3L5 → [(A-α-PMo_9_O_31_)_2_(L5)_3_]^6−^ + 3pyridine was −23.2 kJ mol^−1^. These results indicate that the formation of II was considerably more favorable than that of [(A-α-PMo_9_O_31_)_2_(L5)_3_]^6−^, which is likely owing to the stronger coordination of imidazole ligands to Mo atoms when compared with pyridine ligands. In addition, we investigated the isomerization from [α-PMo_9_] to [β-PMo_9_] units in the aforementioned hybrids. The standard Gibbs energies for isomerization from [α-PMo_9_] to [β-PMo_9_] units in [(A-α-PMo_9_O_31_)_2_(L5)_3_]^6−^ and II were 15.58 and 0.38 kJ mol^−1^, respectively. These results support the partial isomerization of [β-PMo_9_] in the imidazole-based II.

**Fig. 5:**
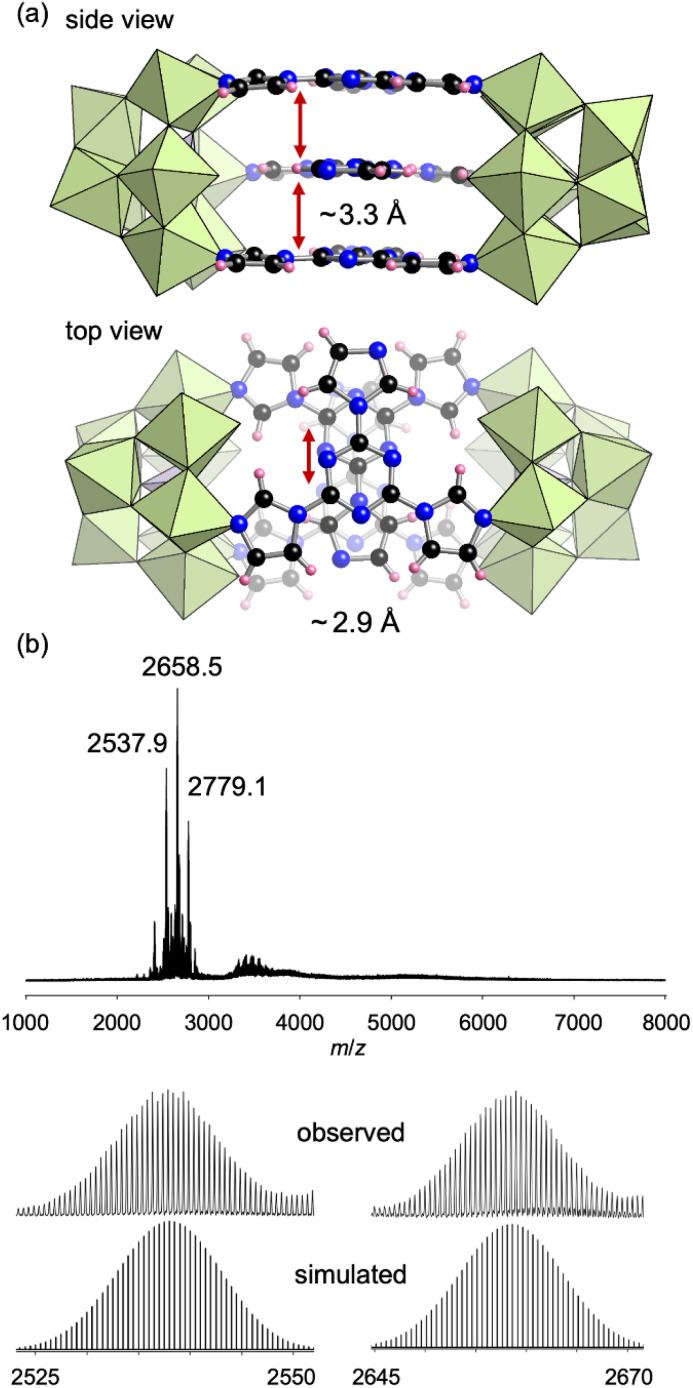
(a) Crystal structure of the anion part of III. The distance between the L3 ligand layers (side view, ∼3.3 Å) and the distance of the centers of the parallel ligands (top view, ∼2.9 Å) are shown. (b) ESI mass spectrum of III in acetonitrile. Inset: enlarged spectra and simulated patterns for *m*/*z* 2537.9 ([TBA_6_H(PMo_9_O_31_)_2_(L3)_3_(CH_3_CN)]^2+^) and 2658.5 ([TBA_7_H(PMo_9_O_31_)_2_(L3)_3_]^2+^).

To construct a more complex structure, we next utilized 2,4,6-(1*H*-imidazol-yl)-1,3,5-triazine (L3), which possesses three imidazole groups at the 1,3,5-positions of the triazine ring, as a ligand. Reacting TBA_3_[A-α-PMo_9_O_31_(pyridine)_3_] and L3 in 2 : 1 ratio in acetonitrile at room temperature (∼25 °C) for 30 min followed by pouring the solution into an excess amount of diethyl ether obtained III as a crude powder. Recrystallization of the crude product in 1,2-dichloroethane obtained single crystals of III suitable for X-ray crystallographic analysis, which revealed that the anion part of III comprised two [PMo_9_] units and three L3 ligands lying in parallel (Fig. 5a, Table S1†). For each L3 ligand, two of three imidazole units coordinated to Mo atoms, and the other imidazole unit remained unreacted. All [PMo_9_] units in the crystalline product of III were [β-PMo_9_] units. The ^31^P NMR spectrum of III in CD_3_CN exhibited a signal at 0.36 ppm, which was attributed to the [β-PMo_9_] isomer (Fig. S5a†). The ESI mass spectrum of III in acetonitrile exhibited a set of signals at *m*/*z* 2537.9, 2658.5, and 2779.1, which could be assigned to [TBA_6_H_2_(PMo_9_O_31_)_2_(L3)_3_)]^2+^, [TBA_7_H(PMo_9_O_31_)_2_(L3)_3_]^2+^, and [TBA_8_(PMo_9_O_31_)_2_(L3)_3_]^2+^, respectively (Fig. 5b). Based on these results as well as elemental analysis, TG-DTA, and ^31^P NMR results (Fig. S2c and S5a†), the formula of III was determined as TBA_6_[(PMo_9_O_31_)_2_(L3)_3_](C_2_H_4_Cl_2_)_2_.

**Fig. 6:**
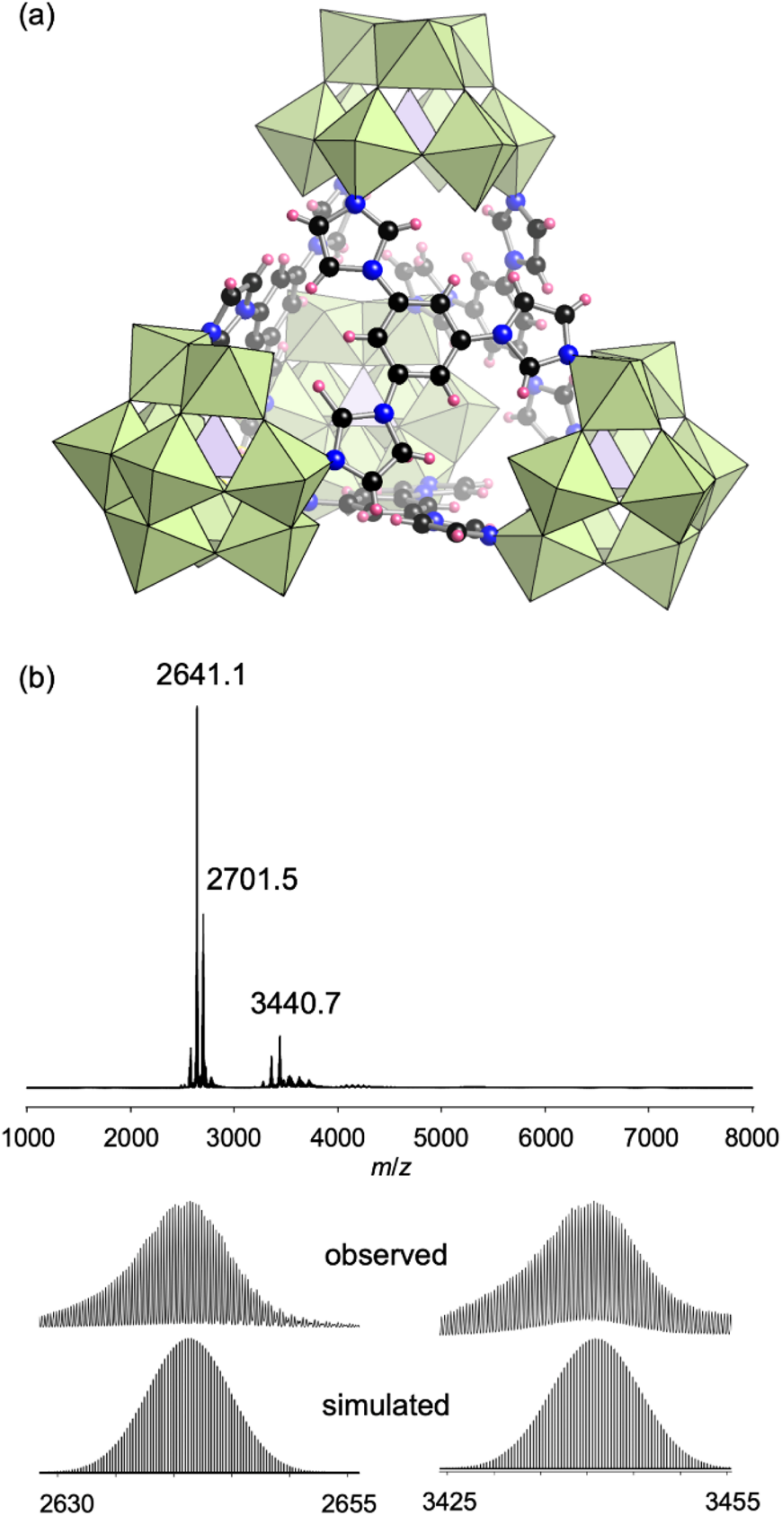
(a) Crystal structure of the anion part of IV. (b) ESI mass spectrum of III in acetonitrile. (Inset): enlarged spectrum and simulated pattern for *m*/*z* 2641.1 ([TBA_16_(PMo_9_O_31_)_4_(L4)_4_(H_2_O)]^4+^) and 3440.7 ([TBA_15_(PMo_9_O_31_)_4_(L4)_4_(H_2_O)]^3+^).

Despite three imidazole groups in L3, the obtained III was a dimer structure in which one of the three imidazole units remained uncoordinated in each ligand. The distance between the L3 ligand layers in III was approximately 3.3 Å and the centers of the parallel ligands were split by approximately 2.9 Å (Fig. 5a), indicating the existence of [π–σ]^2^ interactions between the three L3 ligands.^12^ To avoid such interactions, we next used 1,3,5-tri(1*H*-imidazol-1-yl)benzene (L4), which possesses a benzene ring instead of a triazine at the center, as a ligand. Reacting L4 with TBA_3_[PMo_9_O_31_(pyridine)_3_] in 2 : 1 ratio in *N*,*N*-dimethylformamide at 50 °C afforded IV as a crude powder. Recrystallization of the crude product in a mixed solvent of *N*,*N*-dimethylacetamide and 1,4-dioxane afforded single crystals of IV. X-ray crystallographic analysis revealed that the anion part of IV was a tetrahedral tetramer cage structure where four [PMo_9_] units and four L4 ligands were placed at the vertices and faces, respectively (Fig. 6a, Table S1†). The ESI mass spectrum of IV in CH_3_CN exhibited signals at *m*/*z* 2641.1, 2701.5, and 3440.7, which could be assigned to [TBA_16_(PMo_9_O_31_)_4_(L4)_4_(H_2_O)]^4+^, [TBA_17_(PMo_9_O_31_)_4_(L4)_4_(OH)]^4+^, and [TBA_15_(PMo_9_O_31_)_4_(L4)_4_(H_2_O)]^3+^, respectively (Fig. 6b).

The tetrahedral structure of IV had an inner cavity, where the distance between the P atom of the [PMo_9_] unit and the center of the opposite ligand L4 was about 12.9 Å (Fig. S6†). Some of the [α-PMo_9_] units were transformed into [β-PMo_9_] units, and the X-ray crystallographic analysis was performed with 1 : 1 ratio of these units. Based on the above results as well as elemental analysis, TG-DTA, and ^31^P NMR spectra (Figs. S2d and S5b†), the formula of IV was determined to be TBA_12_[(PMo_9_O_31_)_4_(L4)_4_](C_4_H_9_NO)_4_.

## Conclusions

We report the first method for synthesizing self-assembled molecular hybrid structures *via* direct coordination of imidazole ligands to lacunary POMs. In particular, using the pyridine-coordinated trivacant lacunary polyoxomolybdate TBA_3_[A-α-PMo_9_O_31_(pyridine)_3_] as a precursor, we successfully synthesized several hybrids: the capped monomer I, pillar dimer II, sandwich-like dimer III, and tetrahedron tetramer IV. Because this synthesis method is applicable to various types of lacunary POMs and imidazole ligands, we expect it to help increase the number of POM–organic hybrids as well as their potential applications, such as in catalysis, photocatalysis, energy conversion, and sensors.

## Data availability

The data supporting this manuscript is available in the ESI† and available on request.

## Author contributions

H. S., A. J., C. L. performed the synthesis and characterizations. H. S., A. J., C. L., K. Yo. performed the crystallographic analysis. A. J. and K. S. performed the DFT calculations. All authors analyzed and discussed the results. K. S. conceived and directed the project. H. S., K. Yo., K. Ya., K. S. co-wrote the manuscript.

## Conflicts of interest

There are no conflicts of interest to declare.

## Supplementary Material

SC-015-D4SC02384F-s001

SC-015-D4SC02384F-s002
