# Supplementary material for: Self-assembled molecular hybrids comprising lacunary polyoxometalates and multidentate imidazole ligands
Source: Chem Sci. 2024 May 16;15(24):9281–6. doi: 10.1039/d4sc02384f (PMC11186312; doi:10.1039/d4sc02384f)
Supplement: SC-015-D4SC02384F-s001 [file SC-015-D4SC02384F-s001.pdf]

Electronic Supplementary Material (ESI) for Chemical Science.
This journal is © The Royal Society of Chemistry 2024

Electronic Supplementary Information (ESI) for

Self-assembled molecular hybrids comprising lacunary polyoxometalates and multidentate imidazole ligands

Haoran Sun,[a] Atsuhiro Jimbo,[a] Chifeng Li,[a] Kentaro Yonesato,[a] Kazuya Yamaguchi[a] and Kosuke Suzuki*,[a]

[a] Department of Applied Chemistry, School of Engineering, The University of Tokyo, 7-3-1 Hongo, Bunkyo-ku, Tokyo 113-8656, Japan.

Table of Contents **Page**

1. Experimental Section S2–S4
2. Supplementary Figures (Figs. S1–S7) S5–S9
3. Supplementary Table (Tables S1) S10
4. References S10

1. Experimental Section

Regents

Diethyl ether, 1,2-dichloroethane, ethyl acetate, acetonitrile, pyridine, *N*,*N*-dimethylformamide, 1,4-dioxane, and acetone were obtained from Kanto Chemical. *N*,*N*-Dimethylacetamide, 1,3,5-tris[(1*H*-imidazol-1-yl)methyl]benzene, 1,4-di(1*H*-imidazol-1-yl)benzene, 1,3,5-tri(1*H*-imidazol-1-yl)benzene were obtained from Tokyo Chemical Industry. 2,4,6-Tri(1*H*-imidazol-1-yl)-1,3,5-triazine was obtained from BLDpharm. TBA_3[A-α-PMo_9O_{31}(pyridine)$_3$] was synthesized according to the procedure in our previous report.[S1]

Instruments

^{1}H and ^{31}P NMR spectra were measured using a JEOL JNM-ECA500 instrument (^{1}H, 500.16 MHz; ^{31}P, 202.47 MHz) in deuterated acetonitrile (CD_3CN) using 5 mm tubes. Chemical shifts of ^{1}H NMR spectra were reported in ppm downfield from tetramethylsilane. Chemical shifts of ^{31}P NMR spectra were reported in ppm upfield from phosphoric acid (solvent, D_2O). ^{31}P NMR spectra were measured without nuclear Overhauser effect and with a sufficiently long relaxation time (10 s). Infra-red (IR) spectra were measured using a JASCO FT/IR-4100 instrument by the KBr pellet method. Electrospray ionization (ESI) mass spectra were measured using a Waters Xevo G2-XS QTof instrument. Thermogravimetry differential thermal analysis (TG-DTA) was conducted using a Rigaku Thermo Plus TG8120 instrument under nitrogen gas flow (100 mL/min). Elemental analysis for P and Mo was performed using a Shimadzu ICPS-8100 instrument. Elemental analysis for C, H, and N was performed using a MICRO CORDER JM10 at the Open Facility Center, Tokyo Institute of Technology.

Single crystal X-ray diffraction analysis

Diffraction measurements of **II**, **III**, and **IV** were performed on the BL02B1 beamline at the SPring-8 facility of the Japan Synchrotron Radiation Research Institute (λ = 0.4142 Å for **II** and **III**, 0.4152 Å for **IV**, monochromatized by a Si(311) double-crystal monochromator) with a PILATUS3 X CdTe 1M detector at −173°C. Diffraction measurements of **I** were performed on a Rigaku XtaLAB Synergy-R diffractometer with rotating anode Mo Kα radiation (λ = 0.71073 Å, 50 kV, 24 mA) at −180°C. Data processing was carried out using CrysAlisPro,[S2] including absorption correction, Lorentz correction, and polarization correction. Structural analyses were performed using Olex2 and WinGX.[S3] Structures were solved using SHELXT-2018/1 (intrinsic phasing methods) and refined by SHELXL-2018/3.[S4] All non-hydrogen atoms (C, N, O, P, Mo, and Cl) were refined anisotropically. Highly disordered TBA ions and solvent molecules were omitted by using SQUEEZE program.[S5] CCDC-2345494 (**I**), -2345495 (**II**), -2345496 (**III**), and -2345497 (**IV**) contain the supplementary crystallographic data. The data can be obtained free from The Cambridge Crystallographic Data Centre via www.ccdc.cam.ac.uk/data_request/cif.

Bond valence sum (BVS) calculations: BVS values were calculated by the expression for the variation of the length r_{ij} of a bond between two atoms *i* and *j* in observed crystal with valence *Vi*:

$$V_i = \sum_j \exp\left(\frac{r_0' - r_{ij}}{B}\right)$$

where *B* is constant equal to 0.37 Å, r'_0 is bond valence parameter for a given atom pair.[S6, S7]

Synthesis and characterization of $TBA_3[(PMo_9O_{31})(L1)](C_2H_4Cl_2)_2$ (I)

1,3,5-Tris((1H-imidazol-1-yl)methyl)benzene (**L1**, 19.0 mg, 60 μmol) was dissolved in acetonitrile (12 mL), and the solution was heated to 80 °C. Then, $TBA_3[A\text{-}\alpha\text{-}PMo_9O_{31}(pyridine)_3]$ (72 mg, 30 μmol) was added into the solution, and the solution was stirred at 80 °C for 10 min. The reaction solution was poured into an excess amount of diethyl ether to obtain a crude powder. The crude powder was dissolved in a mixed solvent of acetonitrile and 1,2-dichloroethane, followed by adding diethyl ether and ethyl acetate to obtain crude crystals. Finally, recrystallization was performed in a mixed solvent of 1,2-dichloroethane and diethyl ether to obtain single crystals of **I** in 4 days (46% yield based on $TBA_3[A\text{-}\alpha\text{-}PMo_9O_{31}(pyridine)_3]$). ^{31}P NMR (CD_3CN): δ = −0.61, −2.06 ppm. ESI mass (CH_3CN, positive mode): *m*/*z* 2437.0 (calculated: 2437.0 for $[TBA_3H(PMo_9O_{31})(\mathbf{L1})]^+$), 2678.3 (calculated: 2678.3 for $[TBA_4(PMo_9O_{31})(\mathbf{L1})]^+$). Elemental analysis calcd. (%) for $TBA_3[(PMo_9O_{31})_2(\mathbf{L1})](C_2H_4Cl_2)_2$: Mo, 32.78; P, 1.18; C, 31.92; H, 5.13; N, 4.79. Found: Mo, 34.91; P, 1.17; C, 32.12; H, 5.13; N, 4.93. IR (KBr pellet, cm^{-1}): 3119, 2961, 2873, 1630, 1523, 1483, 1381, 1285, 1237, 1152, 1108, 1094, 1057, 1006, 930, 915, 894, 867,841, 764, 714, 654, 624, 596, 569, 518, 493, 435, 410, 380, 372, 348, 334, 311.

Synthesis and characterization of $TBA_{5.7}H_{0.3}[(PMo_9O_{31})_2(L2)_3](C_2H_4Cl_2)$ (II)

1,4-Di(1H-imidazol-1-yl)benzene (**L2**, 19.2 mg, 90 μmol) was dissolved in acetonitrile (12 mL), and the solution was heated to 50 °C. Then, $TBA_3[A\text{-}\alpha\text{-}PMo_9O_{31}(pyridine)_3]$ (72 mg, 30 μmol) was added into the solution, and the solution was stirred at 50 °C for 10 min. The reaction solution was poured into an excess amount of diethyl ether to obtain the crude powder. The crude powder was recrystallized in 1,2-dichloroethane to obtain single crystals of **II** in a week (25% yield based on $TBA_3[A\text{-}\alpha\text{-}PMo_9O_{31}(pyridine)_3]$). ^{31}P NMR (CD_3CN): δ = –0.01, –1.34 ppm. ESI mass (CH_3CN, positive mode): *m*/*z* 2434.0, (calculated: 2434.0 for $[TBA_6H_2(PMo_9O_{31})_2(\mathbf{L2})_3]^{2+}$). Elemental analysis calcd. (%) for $TBA_{5.7}H_{0.3}[(PMo_9O_{31})_2(\mathbf{L2})_3](C_2H_4Cl_2)$: Mo, 35.30; P, 1.27; C, 31.71; H, 4.93; N, 5.07. Found: Mo, 35.67; P, 1.27; C, 31.94; H, 5.16; N, 4.96. IR (KBr pellet, cm^{-1}): 3131, 2961, 2873, 1630, 1534, 1484, 1380, 1325, 1308, 1267, 1254, 1144, 1106, 1055, 1007, 931, 916, 895, 868, 840, 763, 714, 652, 622, 594, 569, 520, 503, 436, 372, 339, 316, 304.

Synthesis and characterization of $TBA_6[(PMo_9O_{31})_2(L3)_3]$ (III)

2,4,6-Tri(1H-imidazol-1-yl)-1,3,5-triazine (**L3**, 16.8 mg, 60 μmol) was dissolved in acetonitrile (12 mL), and $TBA_3[A\text{-}\alpha\text{-}PMo_9O_{31}(pyridine)_3]$ (72 mg, 30 μmol) was added into the solution. After stirring for 30 min at room temperature (~25 °C), the solution was poured into an excess amount of diethyl ether to obtain crude powder. The crude powder was recrystallized in 1,2-dichloroethane to obtain single crystals of **III** in 10 days (28% yield based on $TBA_3[A\text{-}\alpha\text{-}PMo_9O_{31}(pyridine)_3]$). ^{31}P NMR (CD_3CN): δ = 0.36 ppm. ESI mass (CH_3CN, positive mode): *m*/*z* 2537.9 (calculated: 2538.0 for $[TBA_6H_2(PMo_9O_{31})_2(\mathbf{L3})_3]^{2+}$), 2658.5 (calculated: 2658.6 for $[TBA_7H(PMo_9O_{31})_2(\mathbf{L3})_3]^{2+}$), and 2779.1 (calculated: 2779.3 for $[TBA_8(PMo_9O_{31})_2(\mathbf{L3})_3]^{2+}$). Elemental analysis calcd. (%) for $TBA_6[(PMo_9O_{31})_2(\mathbf{L3})_3]$: Mo, 32.92; P, 1.18; C, 30.76; H, 4.94; N, 8.78. Found: Mo, 32.91; P, 1.08; C, 30.35; H, 4.94; N, 8.79. IR (KBr pellet, cm^{-1}): 3108, 2961, 2873, 1624, 1584, 1550, 1483, 1458, 1451, 1381, 1342, 1314, 1245, 1206, 1152, 1096, 1068, 1047, 1003, 931, 919, 899, 870, 847, 814, 785, 702, 649, 611, 592, 522, 512, 500, 494, 455, 381, 368, 337, 319, 309.

Synthesis and characterization of $TBA_{12}[(PMo_9O_{31})_4(L4)_4](C_4H_9NO)_4$ (IV)

1,3,5-Tri(1H-imidazol-1-yl)benzene (**L4**, 16.5 mg, 60 μmol) was dissolved in *N,N*-dimethylformamide (12 mL), and the solution was heated to 50 °C. Then, $TBA_3[A\text{-}\alpha\text{-}PMo_9O_{31}(pyridine)_3]$ (72 mg, 30 μmol) was added into

the solution, and the solution was stirred at 50 °C for 15 min. The reaction solution was poured into an excess amount of diethyl ether to obtain the crude powder. The powder was recrystallized in a mixed solvent of *N*,*N*-dimethylacetamide and 1,4-dioxane to obtain crude crystals. Finally, recrystallization of the crude crystals in a mixed solvent of *N*,*N*-dimethylacetamide and 1,4-dioxane afforded single crystals of **IV** in 3 days (33% yield based on $TBA_3[A\text{-}\alpha\text{-}PMo_9O_{31}(pyridine)_3]$). ESI mass ($CH_3CN$, positive mode): *m*/*z* 2641.1 (calculated: 2641.2 for $[TBA_{16}(PMo_9O_{31})_4(\mathbf{L4})_4(H_2O)]^{4+}$), 2701.5 (calculated: 2701.5 for $[TBA_{17}(PMo_9O_{31})_4(\mathbf{L4})_4(OH)]^{4+}$), 3440.7 (calculated: 3340.9 for $[TBA_{15}(PMo_9O_{31})_4(\mathbf{L4})_4(H_2O)]^{3+}$). Elemental analysis calcd. (%) for $TBA_{12}[(PMo_9O_{31})_4(\mathbf{L4})_4](C_4H_9NO)_4$: Mo, 34.80; P, 1.25; C, 32.43; H, 5.24; N, 5.64. Found: Mo, 34.88; P, 1.17; C, 32.34; H, 5.92; N, 5.23. IR (KBr pellet, cm^{-1}): 3123, 2960, 2872, 1677, 1623, 1541, 1522, 1483, 1381, 1316, 1267, 1155, 1108, 1082, 1051, 1005, 930, 914, 873, 834, 768, 716, 622, 593, 568, 517, 497, 438, 408, 398, 379, 371, 336, 324, 310.

DFT calculations

DFT calculations were performed using Gaussian 16, Rev. B.01. The geometries used in the calculation were based on the crystal structures determined in this study. The geometry optimizations of the anion structures were optimized at the CAM-B3LYP functional with 6-31G(d) for Si, 6-31+G(d) for C, H, N, O, and LanL2DZ for W by using the polarizable continuum model with the parameters of the integral equation formalism model for acetonitrile. After the geometry optimizations, the standard Gibbs energy of reaction ($\Delta_r G°$) was obtained by frequency calculations.

2. Supplementary Figures

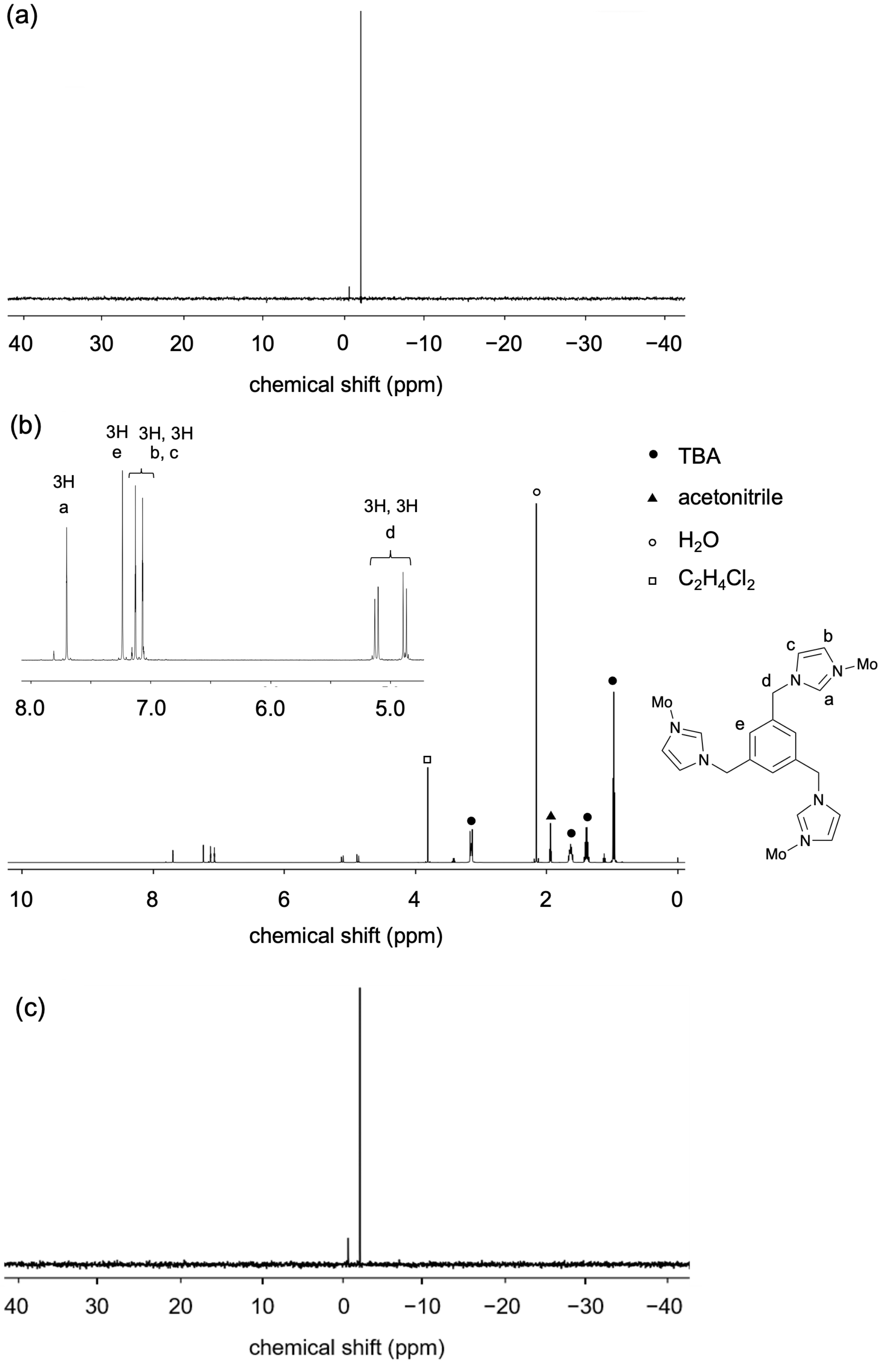

Fig. S1 (a) ^{31}P and (b) ^{1}H NMR spectra of **I** in CD_3CN. (c) ^{31}P NMR spectrum of **I** in CD_3CN after 3 days.

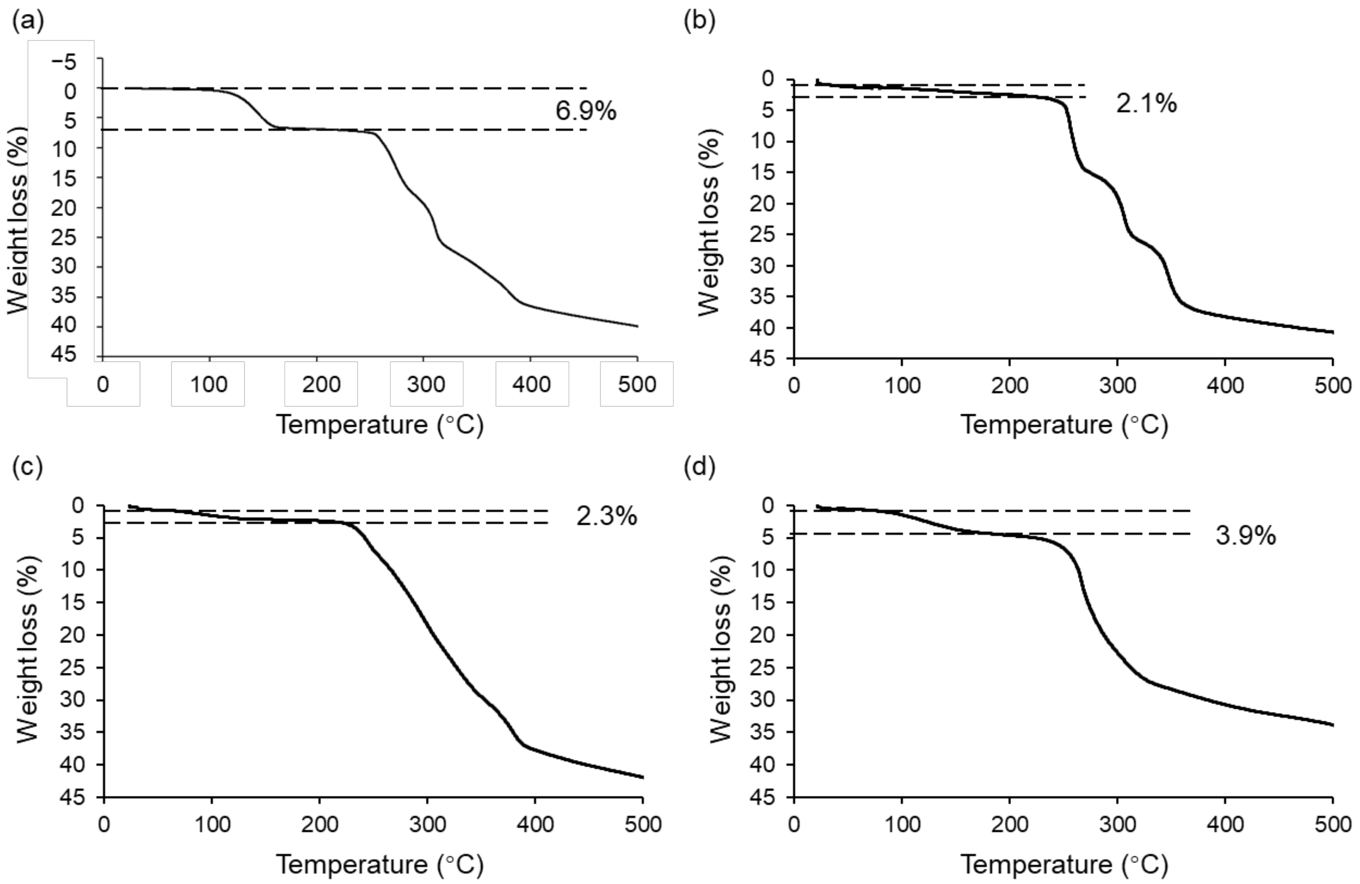

Fig. S2 TG-DTA of (a) **I**, (b) **II**, (c) **III**, and (d) **IV**.

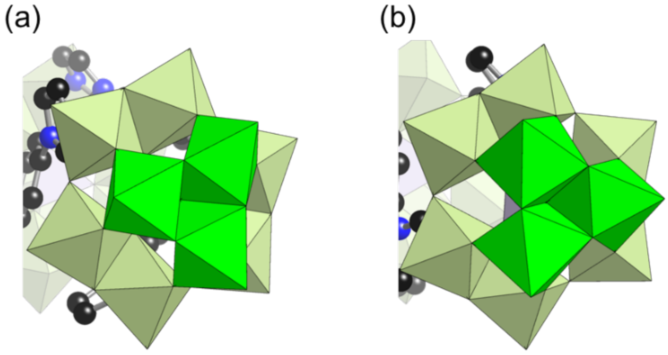

Fig. S3 Enlarged views of (a) [α-PMo_9] unit and (b) [β-PMo_9] units in **II**. Light green and light purple polyhedra represent [MoO_6] and [PO_4], respectively. Blue and black spheres represent N and C, respectively. Green polyhedra represent [Mo_3O_{13}] units that show the difference between [α-PMo_9] and [β-PMo_9] units with 60° rotation.

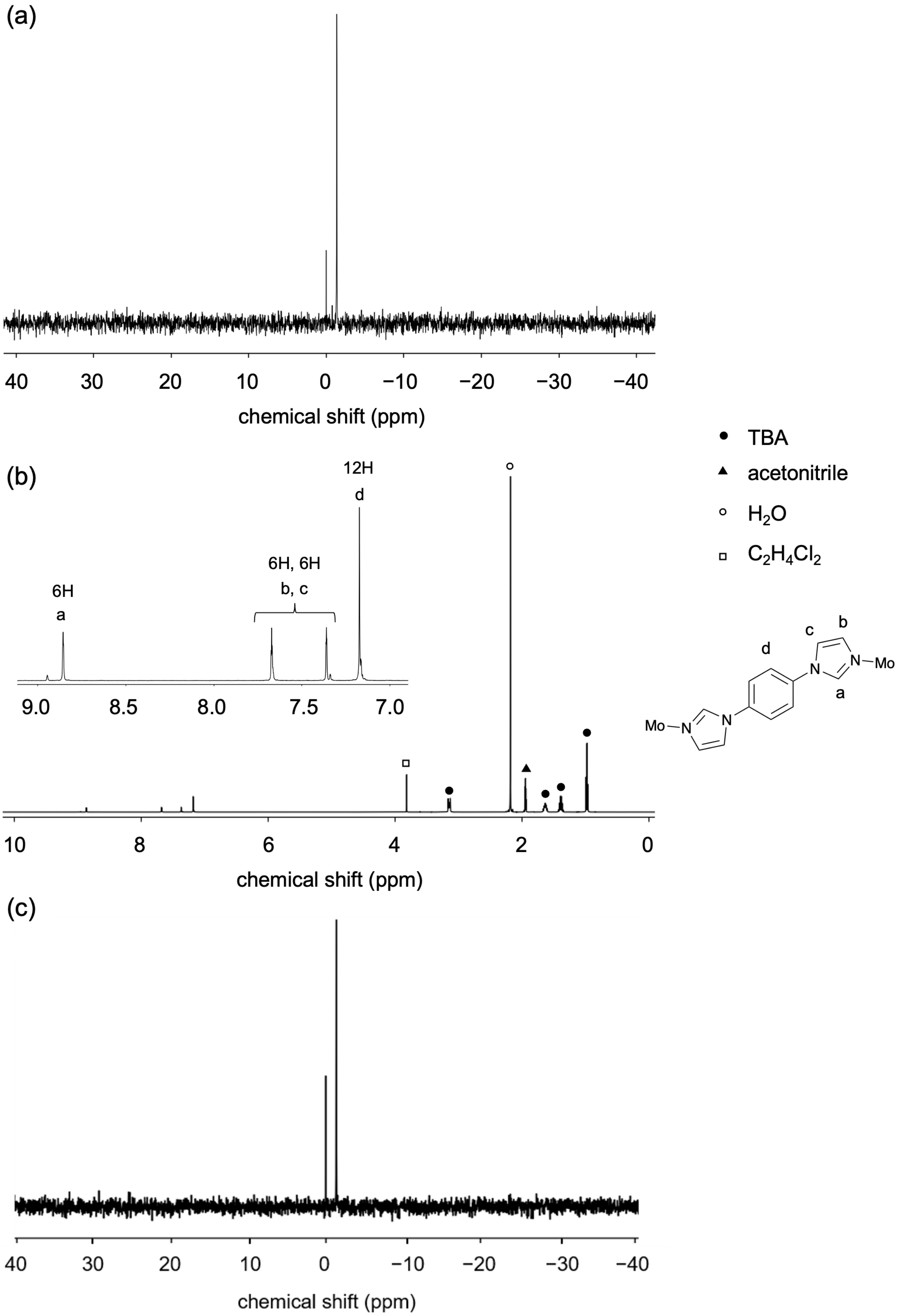

Fig. S4 (a) ^{31}P and (b) ^{1}H NMR spectra of **II** in CD_3CN. (c) ^{31}P NMR spectrum of **II** in CD_3CN after 3 days.

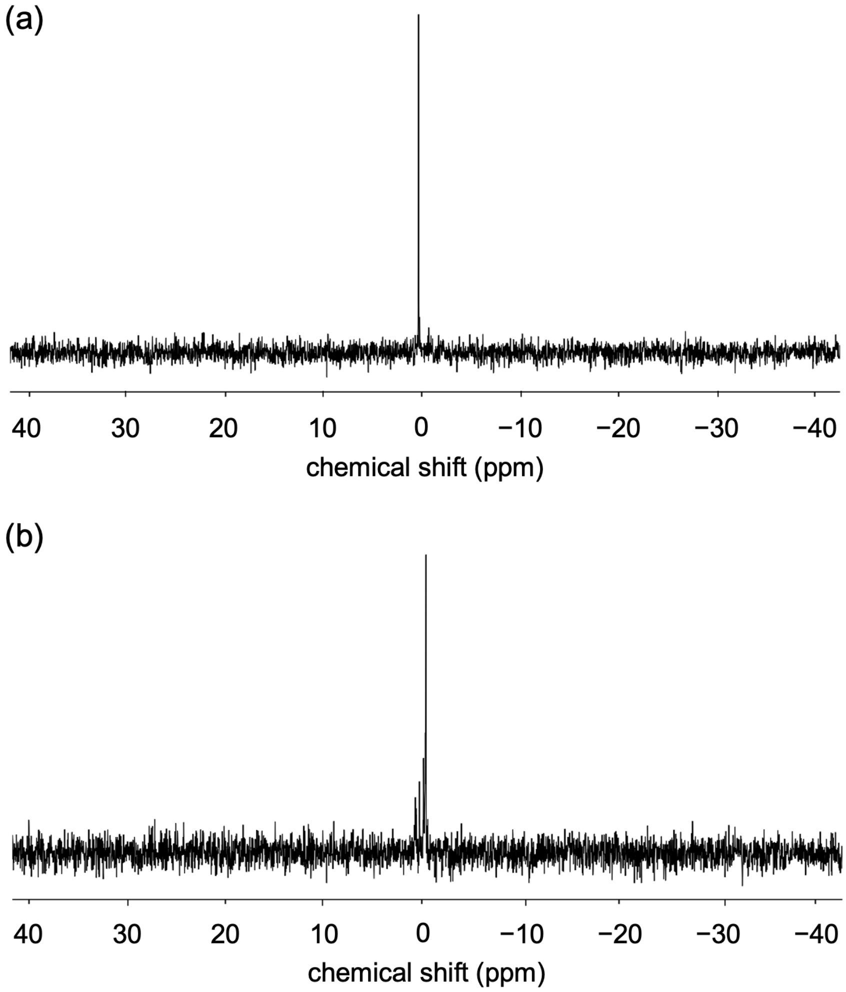

Fig. S5 ^{31}P NMR spectra of (a) **III** and (b) **IV** in CD_3CN.

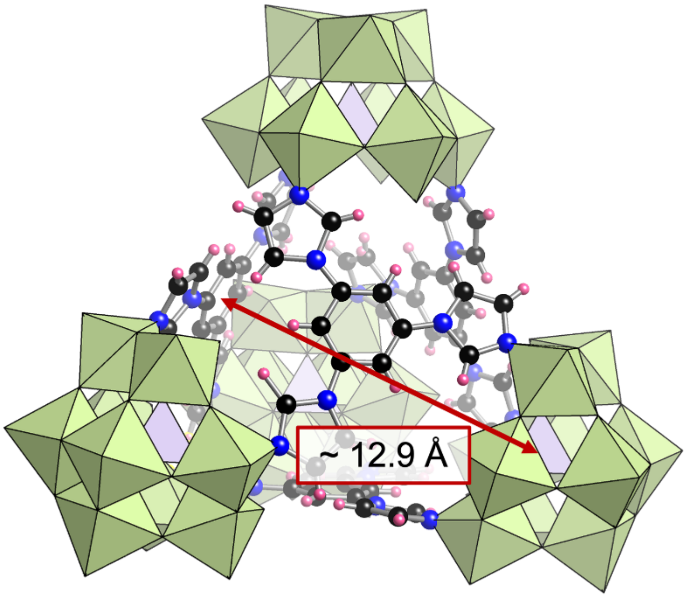

Fig. S6 (a) Structure of $[(PMo_9O_{31})_2(\mathbf{L3})_3]^{6-}$ (**IV**). The distance between the P atom of the [PMo₉] unit and the center of the opposite ligand **L4** is shown (~12.9 Å).

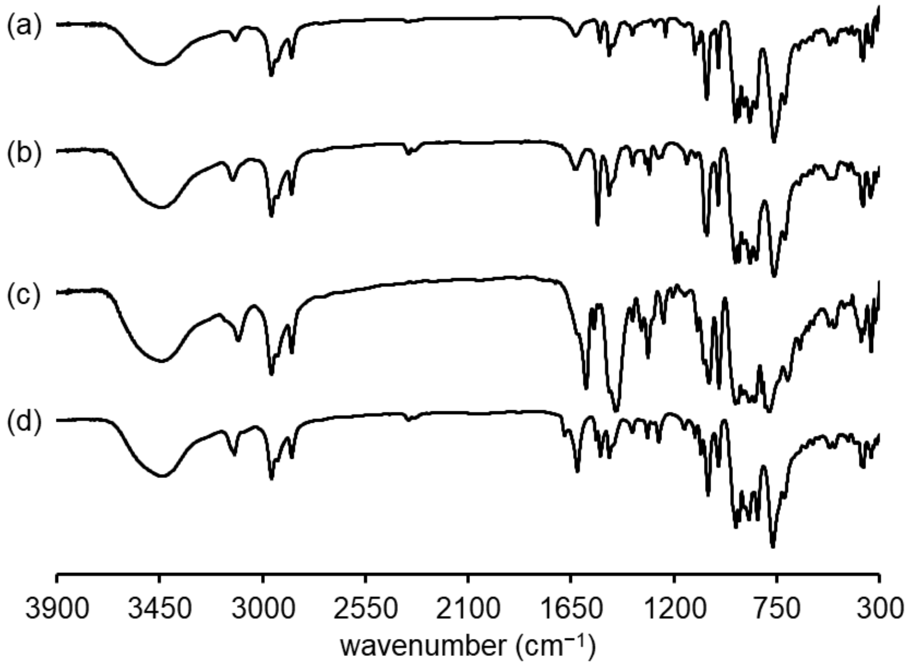

Fig. S7 IR spectra of (a) **I**, (b) **II**, (c) **III**, and (d) **IV**.

3. Supplementary Table

Table S1. Crystal data of **I**, **II**, **III**, and **IV**.

	**I**	**II**	**III**	**IV**
Formula	$C_{70}H_{134}Cl_4Mo_9N_9O_{31}P$	$C_{92}H_{154}Cl_8Mo_{18}N_{15}O_{62}P_2$	$C_{120}H_{215}Cl_4Mo_{18}N_{32}O_{62}P_2$	$C_{60}H_{48}Mo_{36}N_{24}O_{124}P_4$
Fw (g/mol)	2634.09	4534.75	5028.89	6666.94
Crystal system	Orthorhombic	Monoclinic	Triclinic	Tetragonal
Space group	*Pbca*	$P2_1/n$	$P\bar{1}$	$I4_1/a$
a (Å)	18.9192(2)	27.1448(3)	18.2063(2)	39.2789(3)
b (Å)	25.1964(4)	27.3825(3)	23.9584(2)	39.2789(3)
c (Å)	43.0574(5)	29.9056(5)	27.3167(8)	32.7662(5)
α (deg)	90	90	85.3618(9)	90
β (deg)	90	100.6111(13)	76.6634(9)	90
γ (deg)	90	90	87.3652(8)	90
V (Å^3)	20525.3(5)	21848.5(5)	11449.7(5)	50552.7(11)
Z	8	4	2	4
ρ_{calc} (g/cm^3)	1.705	1.379	1.459	1.306
Temp. (K)	93(2)	100(2)	100(2)	100(2)
GOF	1.033	1.056	1.083	1.073
R_1 [$I > 2\sigma(I)$]	0.0540	0.0645	0.0644	0.0641
wR_2	0.1583	0.1922	0.1999	0.2196

$R_1 = \Sigma||F_o|-|F_c||/\Sigma|F_o|$, $wR_2 = (\Sigma[w(F_o^2 - F_c^2)]/\Sigma[w(F_o^2)^2])^{1/2}$

4. References

S1 C. Li, N. Mizuno, K. Yamaguchi and K. Suzuki, *J. Am. Chem. Soc.*, 2019, **141**, 7687.

S2 Rigaku OD. CrysAlis PRO. Rigaku Oxford Diffraction Ltd, Yarnton, England (2018).

S3 L. J. Farrugia, *J. Appl. Crystallogr.*, 1999, **32**, 837.

S4 (a) G. M. Sheldrick, *Acta Crystallogr.*, 2008, **A64**, 112; (b) G. M. Sheldrick, *Acta Crystallogr.*, 2015, **C71**, 3.

S5 P. van der Sluis andA. L. Spek, *Acta Crystallogr.*, 1990, **A46**, 194.

S6 N. E. Brese and M. O'Keeffe, *Acta Crystallogr.*, 1991, **B47**, 192.

S7 I. D. Brown and D. Altermatt, *Acta Crystallogr.*, 1985, **B41**, 244.
